# lncRNA GPRC5D-AS1 as a ceRNA inhibits skeletal muscle aging by regulating miR-520d-5p

**DOI:** 10.18632/aging.205279

**Published:** 2023-12-09

**Authors:** Miao Yu, Xiuting He, Ting Liu, Jie Li

**Affiliations:** 1 Department of Geriatrics and Special Medical Treatment, The First Hospital of Jilin University, Changchun 130021, China

**Keywords:** long non-coding RNA, miR-520d-5p, skeletal muscle aging, GPRC5D-AS1, competing endogenous RNA

## Abstract

Sarcopenia induced by muscle aging is associated with negative outcomes in a variety of diseases. Long non-coding RNAs are a class of RNAs longer than 200 nucleotides with lower protein coding potential. An increasing number of studies have shown that lncRNAs play a vital role in skeletal muscle development. According to our previous research, lncRNA GPRC5D-AS1 is selected in the present study as the target gene to further study its effect on skeletal muscle aging in a dexamethasone-induced human muscle atrophy cell model. As a result, GPRC5D-AS1 functions as a ceRNA of miR-520d-5p to repress cell apoptosis and regulate the expression of muscle regulatory factors, including MyoD, MyoG, Mef2c and Myf5, thus accelerating myoblast proliferation and differentiation, facilitating development of skeletal muscle. In conclusion, lncRNA GPRC5D-AS1 could be a novel therapeutic target for treating sarcopenia.

## INTRODUCTION

There is an aging population around the world. Aging is a complex phenomenon that refers to the process of physical deterioration as the population ages [1]. As a major organ of body movement and metabolism, skeletal muscle is also highly plastic [2]. Its mass decreases as a result of many factors, including aging, immobilization, diseases and malnutrition [3, 4]. In the elderly, muscle mass decreases causing sarcopenia, functional impairment, tissue disorganization, loss of mass and disability [5, 6], which also increases morbidity and mortality [7]. Sarcopenia mainly occurs in the elderly because of aging [8, 9], which is the leading cause of frailty among elders [10]. It is characterized by progressive and generalized loss of muscles, strength, and function, resulting in fracture, physical disability, and death [11, 12]. In this condition, muscle mass and strength gradually decrease, leading to an increased risk of falls and permanent disability [13]. It is also becoming an increasingly important health concern worldwide [14]. Approximately 5%–13% of people aged 60 or over suffer from sarcopenia, while 50% of people aged 80 or older suffer from the disease [15, 16].

As a complex process, skeletal muscle aging is affected by multiple signaling pathways [17]. The regulation of the process involves the participation of myogenic regulatory factors (MRFs), such as myogenin (MyoG) and myogenic factor 5 (Myf5), as well as myogenic differentiation D (MyoD) [18–20]. These MRFs, in conjunction with their co-regulator, myocyte enhancer factor 2C (MEF2C), play significant roles in the process of myogenesis. The expression of MRFs is controlled by Wingless-type (Wnt) signals [21]. Specifically, Wnt5a, a member of the Wnt family, is responsible for regulating MyoD and Myf5 during myogenesis [22, 23]. However, skeletal muscle myogenesis and differentiation remain poorly understood molecular mechanisms.

Long non-coding RNAs (lncRNAs) are a subclass of RNA molecules exceeding 200 nucleotides in length, exhibiting limited potential for protein coding [24, 25]. These lncRNAs are situated within intergenic regions, distinct from annotated coding genes [26]. Over the past decade, mounting evidence has demonstrated that lncRNAs play crucial roles in various significant biological processes, such as cell fate determination, cellular differentiation, regulation of the cell cycle and proliferation, apoptosis, and aging, through their interactions with essential proteins [27–29]. Notably, a strong association has been observed between the expression of lncRNAs and muscle proliferation, differentiation, and atrophy. For instance, the upregulation of the long non-coding RNA (lncRNA) Atrolnc-1 in catabolic conditions has been observed to enhance muscle atrophy by augmenting NF-κB activity [30]. Similarly, the lncRNA Chronos has been found to be upregulated with advancing age, and its inhibition has been shown to induce myofiber hypertrophy both *in vitro* and *in vivo* [31]. Furthermore, other lncRNAs such as Neat1, Malat1, Sra, and Meg3 exhibit distinct expression patterns during myoblast differentiation, suggesting their crucial involvement in muscle fiber development and maturation [32]. Several studies have proposed that lncRNAs serve as microRNA sponges, thereby playing significant roles in various vital cellular processes, but there is not much information available on whether competing endogenous RNAs (ceRNAs) play a role in muscle aging [29, 33].

As a result, it is imperative to investigate the molecular mechanisms by which lncRNA involves in aging-related diseases in order to combat the problem in today’s aging society. Our earlier report identified that three candidate lncRNAs (GPRC5D-AS1, AC004797.1 and PRKG1-AS1) might play vital roles during the aging process of skeletal muscles [2]. In this study, lncRNA GPRC5D-AS1 was selected as the target gene to further study its effect on skeletal muscle aging in a dexamethasone-induced human muscle atrophy cell model. Currently, most studies on lncRNA profiling have used aged mouse models to date. We utilized a dexamethasone-induced human muscle atrophy cell model, which was considered to be a relatively uncommon approach. Consequently, the identification and functional characterization of lncRNAs associated with muscle aging in this study holds great promise for the development of innovative therapeutic interventions. This investigation presents a potentially groundbreaking strategy for addressing sarcopenia.

## RESULTS

### lncRNA GPRC5D-AS1 restored the proliferation level of atrophic myoblasts

**Figure 1:**
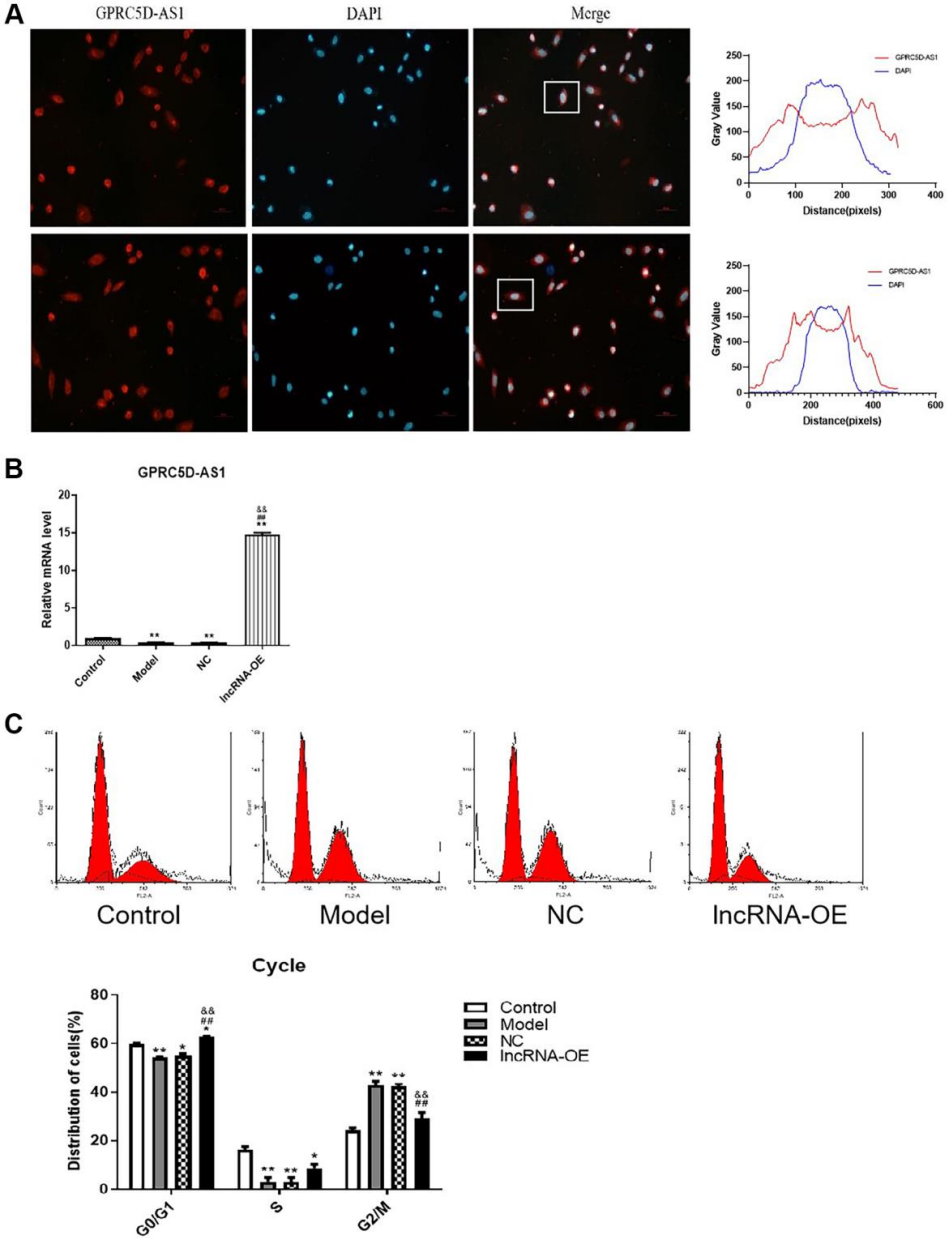
**GPRC5D-AS1 restored the proliferation level of atrophic myoblasts.** (**A**) FISH assay was utilized to identify the subcellular localization of long non-coding RNA (lncRNA) GPRC5D-AS1 in cells (200 ×). The red fluorescence represents GPRC5D-AS1, and the blue fluorescence represents the cell nucleus. Quantification of fluorescence intensities (Gray Value) by ImageJ software. (**B**) The efficiency of overexpression vector encoding GPRC5D-AS1 was examined by qRT-PCR. Human skeletal muscle myoblasts (HSMM) were the control group. 15 mM Dexamethasone (Dex) was added in HSMM to establish atrophy cell model (model group). Empty plasmid (NC group) and GPRC5D-AS1-OE plasmid (lncRNA-OE group) were transfected into atrophy cell model. Differences among groups were analyzed using ANOVA with Bonferroni’s multiple comparison test. ^*^P < 0.05, ^**^P < 0.01 compared with control group; ^#^*P* < 0.05, ^##^*P* < 0.01 compared with model group; ^&^*P* < 0.05, ^&&^*P* < 0.01 compared with NC group. (**C**) Effects of GPRC5D-AS1 overexpression on cell cycle progression using flow cytometry after propidium iodide staining. Representative images were shown. ^*^P < 0.05, ^**^P < 0.01 compared with control group; ^#^*P* < 0.05, ^##^*P* < 0.01 compared with model group; ^&^*P* < 0.05, ^&&^*P* < 0.01 compared with NC group.

In our previous report, we conducted lncRNA sequencing on skeletal muscle samples obtained from elderly and young individuals, and found that lncRNA GPRC5D-AS1 was significantly decreased during skeletal muscle aging process. Furthermore, we validated our findings using clinical samples and a dexamethasone-induced human muscle atrophy cell model [2]. In this study, we further studied its effect on skeletal muscle aging to characterize underlying mechanisms. FISH analysis demonstrated that GPRC5D-AS1 exhibited predominant expression in the cytoplasmic region (Figure 1A). In order to investigate the role of GPRC5D-AS1 on myoblasts, we constructed an overexpressing vector containing GPRC5D-AS1. The efficiency of overexpression was examined by qRT-PCR (Figure 1B).

The proliferation of cells is controlled by the cell cycle, which consists of various phases [34, 35]. Analysis of the cell cycle demonstrated the decreases in the proportion of G0/G1 and S phases, and an increase in the proportion of G2/M phase in the atrophied skeletal muscle myoblasts (Figure 1C and Supplementary Table 1). Notably, the overexpression of GPRC5D-AS1 in model cells resulted in a significant increase in cells in the G0/G1 phase and a decrease in cells in the G2/M phase, which suggested that overexpression of GPRC5D-AS1 restored the cell cycle of the atrophic cells to a situation similar to that of the control group. These findings suggested that GPRC5D-AS1 potentially exerted a positive influence on the regulation of the transition in the skeletal muscle myoblast cycle.

### Overexpression of GPRC5D-AS1 enhanced cell viability and reduced cell apoptosis in atrophy cell

**Figure 2:**
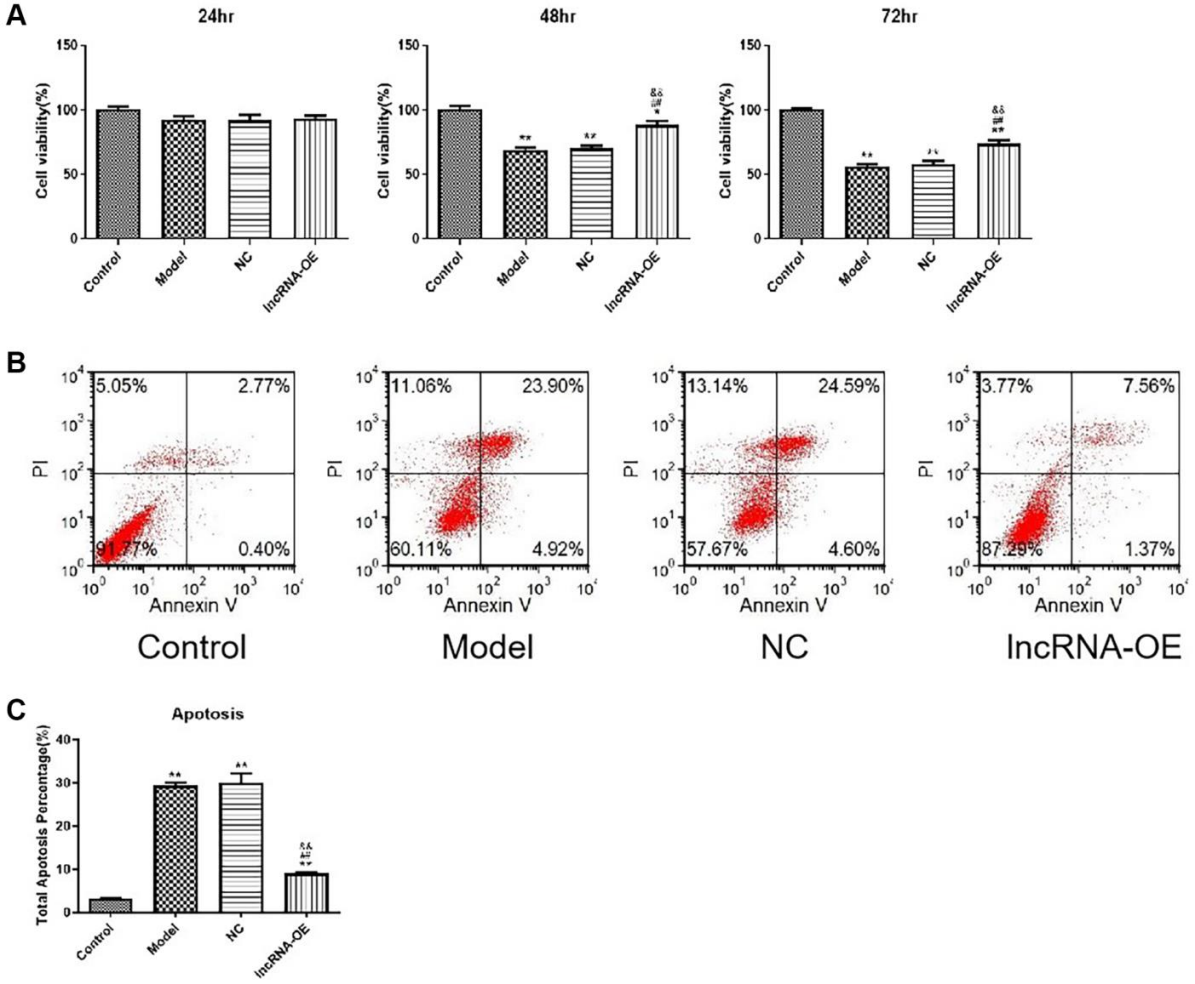
**The effect of overexpression of GPRC5D-AS1 on cell viability and cell apoptosis.** (**A**) Cell viability was assessed by CCK-8 assay. HSMM was control group. Dex (15 mM) was added in HSMM to establish atrophy cell model (model group). Empty plasmid (NC group) and GPRC5D-AS1-OE plasmid (lncRNA-OE group) were transfected into atrophy cell model and incubated for 24 h, 48 h and 72 h. ^*^P < 0.05, ^**^P < 0.01 compared with control group; ^#^*P* < 0.05, ^##^*P* < 0.01 compared with model group; ^&^*P* < 0.05, ^&&^*P* < 0.01 compared with NC group. (**B**) Cell apoptosis was assessed by flow cytometry. Groups were set as previously mentioned. Empty plasmid and GPRC5D-AS1-OE plasmid were transfected into atrophy cell model and incubated for 48 h. (**C**) Quantitative analysis of cell apoptosis. ^*^P < 0.05, ^**^P < 0.01 compared with control group; ^#^*P* < 0.05, ^##^*P* < 0.01 compared with model group; ^&^*P* < 0.05, ^&&^*P* < 0.01 compared with NC group.

Next, we investigated whether overexpression of GPRC5D-AS1 influenced cell proliferation and cell apoptosis. In order to measure cell viability, CCK-8 assays were performed at different time points. As determined by the CCK-8 assay, significant reductions in cell viability were observed in the atrophied skeletal muscle myoblasts compared with the control group at 48 and 72 hours. Notably, overexpression of GPRC5D-AS1 could significantly increase cell viability, which reversed reduction to varying degrees in model cells (Figure 2A).

Besides, apoptosis rates were significantly high in the model group based on flow cytometric detection of apoptotic cells. The apoptotic rate of the overexpression group presented a drastic decline in comparison with model group, which suggested that GPRC5D-AS1 overexpression could inhibit atrophy cell apoptosis (Figure 2B). The quantitative analysis of the results of cell apoptosis was shown in Figure 2C.

### Overexpression of GPRC5D-AS1 influenced the expression level of muscle regulatory factors

**Figure 3:**
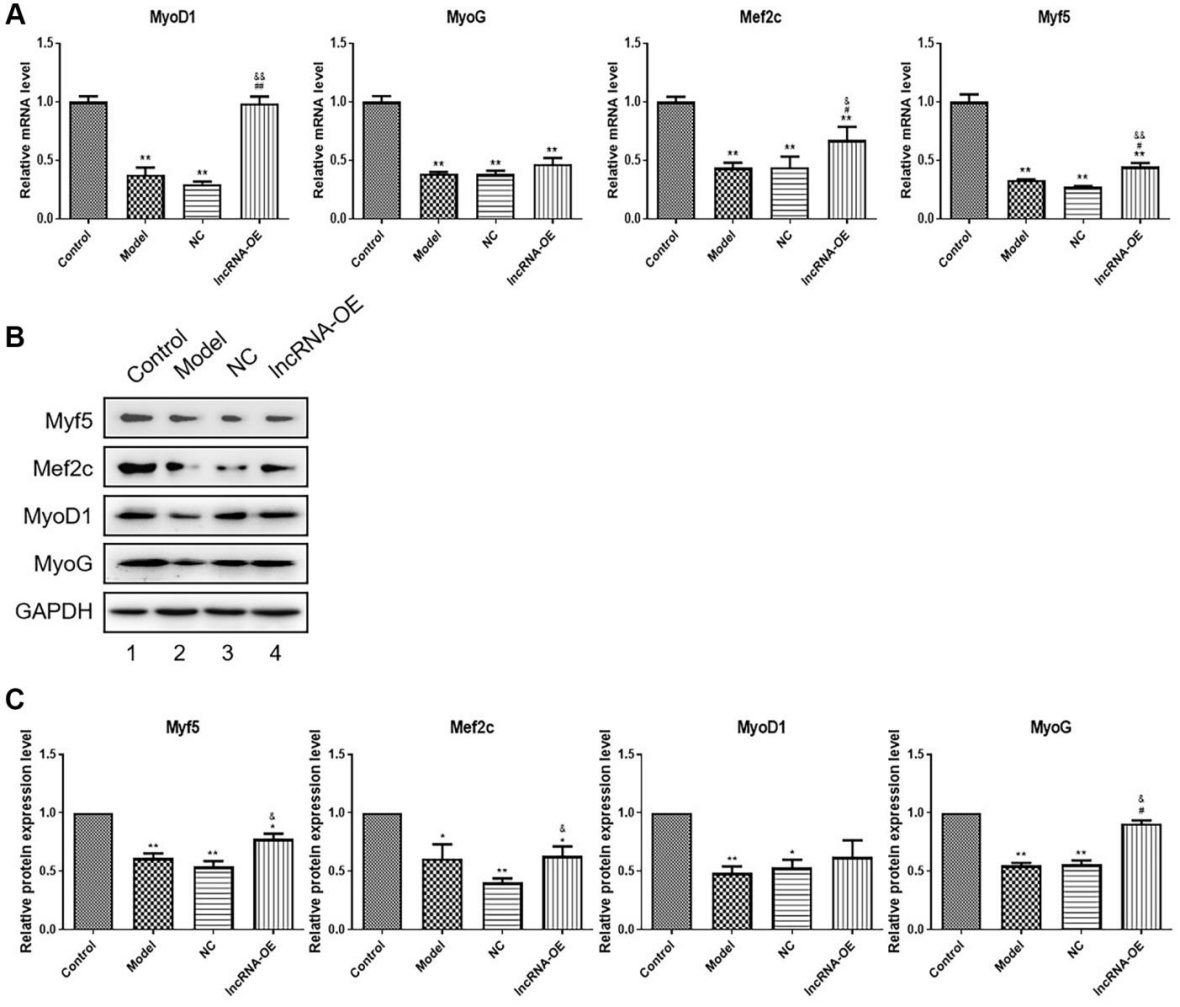
**The effect of overexpression of GPRC5D-AS1 on muscle regulatory factors.** (**A**) qRT-PCR analyzed gene expression of Myf5, MyoG, MyoD and Mef2c. HSMM was control group. Dex (15 mM) was added in HSMM to establish atrophy cell model (model group). Empty plasmid (NC group) and GPRC5D-AS1-OE plasmid (lncRNA-OE group) were transfected into atrophy cell model and incubated for 48 h. ^*^P < 0.05, ^**^P < 0.01 compared with control group; ^#^*P* < 0.05, ^##^*P* < 0.01 compared with model group; ^&^*P* < 0.05, ^&&^*P* < 0.01 compared with NC group. (**B**) Protein expression of Myf5, MyoG, MyoD and Mef2c detected by Western blot. Groups were set as previously mentioned. Empty plasmid and GPRC5D-AS1-OE plasmid were transfected into atrophy cell model and incubated for 48 h. (**C**) Quantitative analysis of western blot. ^*^P < 0.05, ^**^P < 0.01 compared with control group; ^#^*P* < 0.05, ^##^*P* < 0.01 compared with model group; ^&^*P* < 0.05, ^&&^*P* < 0.01 compared with NC group.

Our experiments validated the effect of GPRC5D-AS1 on myogenesis and differentiation in skeletal muscle by detecting the levels of MyoD1, Myf5, Mef2c and MyoG mRNA and protein expression. Based on Figure 3, the dexamethasone-induced muscle atrophy cell model showed significant decreases in these four factors. Their expression at mRNA level were enhanced by transfection with GPRC5D-AS1-OE (Figure 3A). The results obtained by Western blot were consistent with qRT-PCR. Western blot analysis indicated that the expression of MyoD1, MyoG, Mef2c and Myf5 were reduced in model group compared with control. And these muscle regulatory factors expression were upregulated in the GPRC5D-AS1-OE group (Figure 3B, 3C). The above results indicated that GPRC5D-AS1 regulated myoblast differentiation.

### Prediction and validation of miR-520d-5p as one of target miRNAs of GPRC5D-AS1

**Figure 4:**
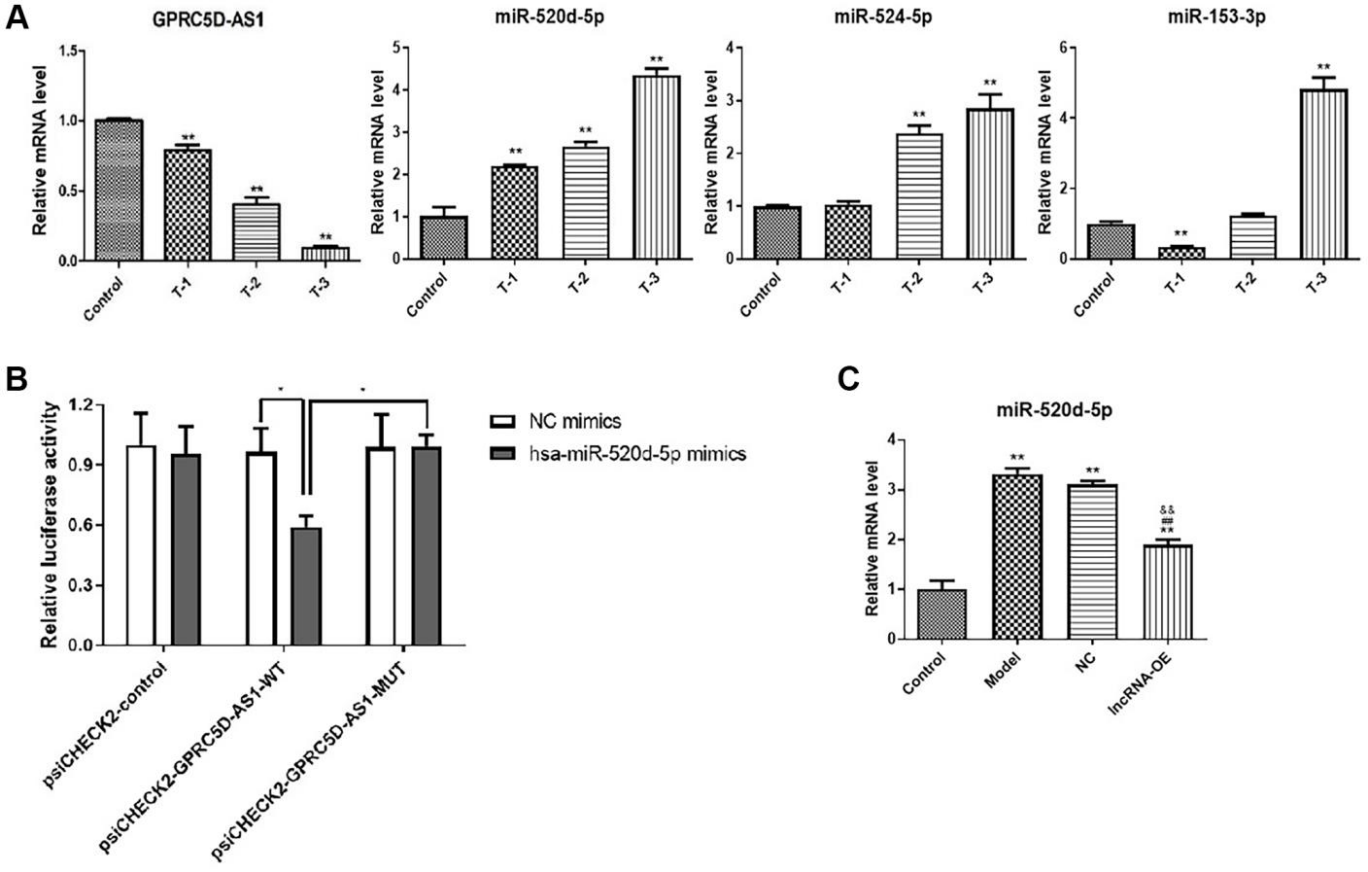
**Prediction and validation of miR-520d-5p as one of target miRNAs of GPRC5D-AS1.** (**A**) qRT-PCR analyzed gene expression level of GPRC5D-AS1, miR-520d-5p, miR-153-3p and miR-524-5p. Different concentrations of Dex (5 mM, 10 mM and 15 mM) were added in HSMM and incubated for 48 h. Control: HSMM; T-1: 5 mM Dex; T-2:10 mM Dex; T-3:15 mM Dex. ^*^P < 0.05, ^**^P < 0.01 compared with control group. (**B**) NC mimic and miR-520d-5p mimic were co-transfected with plasmid psiCHECK2-GPRC5D-AS1-WT luciferase vector or psiCHECK2-GPRC5D-AS1-MUT vector into in human skeletal muscle myoblasts, and the normalized relative luciferase activities (Renilla/firefly) were analyzed. ^*^P < 0.05. (**C**) qRT-PCR analyzed gene expression of miR-520d-5p. HSMM was control group. Dex (15 mM) was added in HSMM to establish atrophy cell model (model group). Empty plasmid (NC group) and GPRC5D-AS1-OE plasmid (lncRNA-OE group) were transfected into atrophy cell model and incubated for 48 h. ^*^P < 0.05, ^**^P < 0.01 compared with control group; ^#^*P* < 0.05, ^##^*P* < 0.01 compared with model group; ^&^*P* < 0.05, ^&&^*P* < 0.01 compared with NC group.

First, we predicted the potential interactive miRNAs of GPRC5D-AS1 using the Starbase database (http://starbase.sysu.edu.cn/index.php), and chose miR-520d-5p, miR-153-3p, miR-524-5p as the candidate miRNAs to qRT-PCR verification. qRT-PCR results indicated that GPRC5D-AS1 expression was down-regulated after dexamethasone-induced human muscle atrophy. The expression of miR-520d-5p, miR-524-5p and miR-153-3p were upregulated to varying degrees. Of these, miR-520d-5p exhibited a marked dose-dependent tendency (Figure 4A).

Then, to further verify whether miR-520d-5p was targeted by GPRC5D-AS1, a luciferase reporter vector was constructed by ligating with GPRC5D-AS1-WT and GPRC5D-AS1-MUT. The findings indicated a significant decrease in luciferase activity in the groups co-transfected with GPRC5D-AS1-WT and miR-520d-5p (Figure 4B). Moreover, we investigated the effect of GPRC5D-AS1 overexpression on the miR-520d-5p. Quantitative real-time PCR result demonstrated that miR-520d-5p significantly upregulated in the dexamethasone-induced muscle atrophy cell model. Its expression was reduced at mRNA level after transfection with GPRC5D-AS1-OE plasmid (Figure 4C). These results provided evidence that miR-520d-5p is among the targeted miRNAs of GPRC5D-AS1.

### lncRNA GPRC5D-AS1 interacted with miR-520d-5p to promote myoblast proliferation

**Figure 5:**
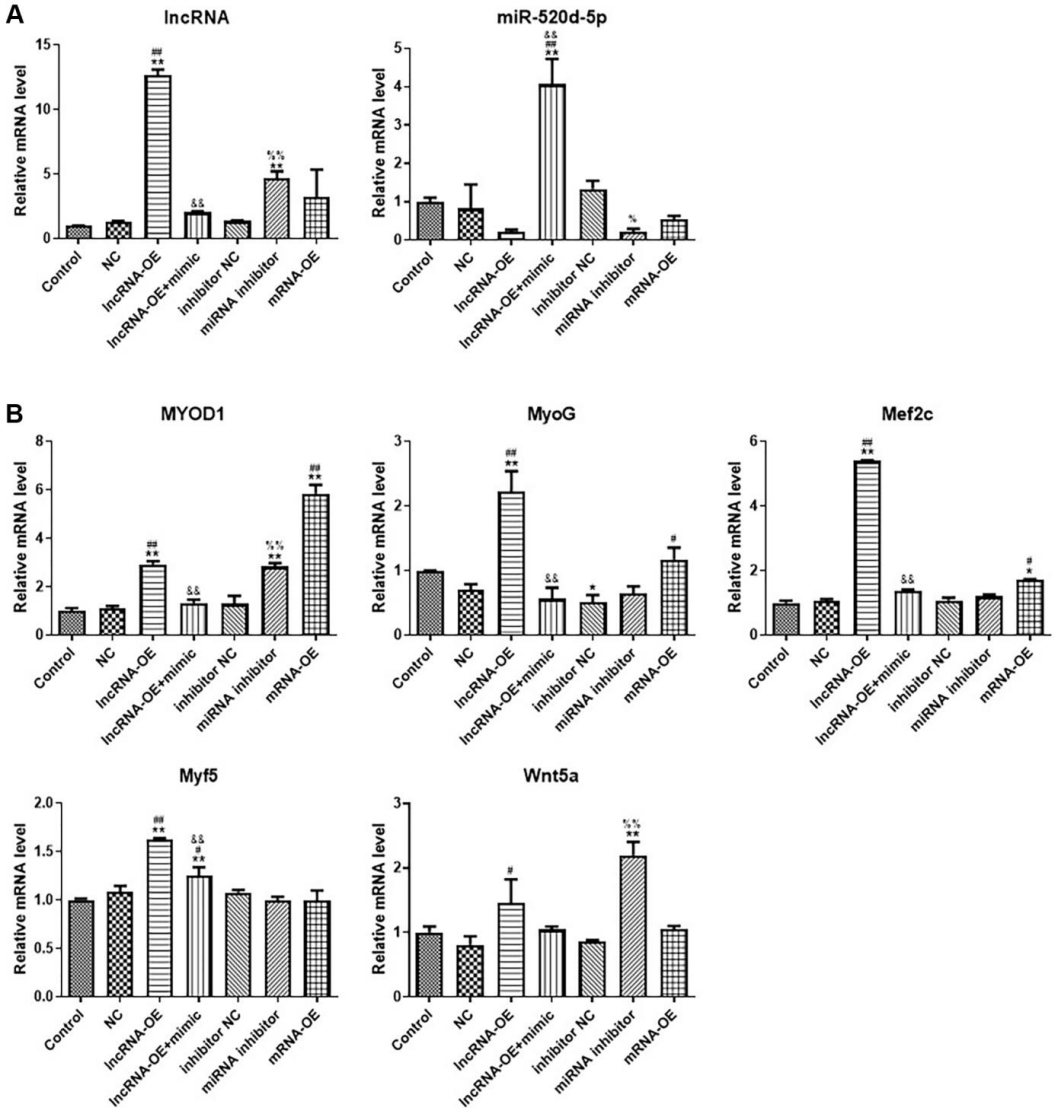
**LncRNA GPRC5D-AS1 interacted with miR-520d-5p to promote myoblast proliferation.** (**A**, **B**) qRT-PCR analyzed gene expression of GPRC5D-AS1, miR-520d-5p, MyoD1, MyoG, Mef2c, Myf5 and Wnt5a. 15 mM Dex was added in human skeletal muscle myoblasts to establish atrophy cell model (control group). Empty plasmid (NC group), GPRC5D-AS1-OE (lncRNA-OE group), GPRC5D-AS1-OE + miR-520d-5p mimic (lncRNA-OE + mimic group), miRNA inhibitor control (inhibitor NC group), miR-520d-5p inhibitor (miRNA inhibitor group) or MYOD1-OE plasmid (mRNA-OE group) was transfected into atrophy cell model and incubated for 48 h. ^*^P < 0.05, ^**^P < 0.01 compared with control group; ^#^*P* < 0.05, ^##^*P* < 0.01 compared with NC group; ^&^*P* < 0.05, ^&&^*P* < 0.01 compared with lncRNA-OE group; ^%^*P* < 0.05, ^%%^*P* < 0.01 compared with inhibitor NC group.

To understand how GPRC5D-AS1 and miR-520d-5p affected atrophy cell myogenesis and differentiation, we treated human muscle atrophy model cells with empty plasmid, GPRC5D-AS1-OE plasmid, MYOD1-OE plasmid, associated miRNA control, miR-520d-5p mimic and inhibition. qRT-PCR was used to verify the transfection effect (Figure 5A). At the same time, we observed that an addition of miR-520d-5p mimic could decrease expression of GPRC5D-AS1. The GPRC5D-AS1 expression was significantly enhanced upon addition of miR-520d-5p inhibitor. The above data indicated the inter-regulation between GPRC5D-AS1 and miR-520d-5p.

By examining the expression of muscle regulatory factors, we found that overexpression of GPRC5D-AS1 increased the expression of MyoD1, MyoG, Mef2c and Myf5, and this phenomenon can be reversed by adding miR-520d-5p mimic, making the expression levels lower. It is noteworthy that miR-520d-5p inhibitor could also enhance MyoD1 and Wnt5a expression. Overexpression of MYOD1 could mildly increase other muscle regulatory factors expression at different degrees, including MyoG and Mef2c (Figure 5B).

### lncRNA GPRC5D-AS1 regulated cell viability and cell apoptosis by miR-520d-5p

**Figure 6:**
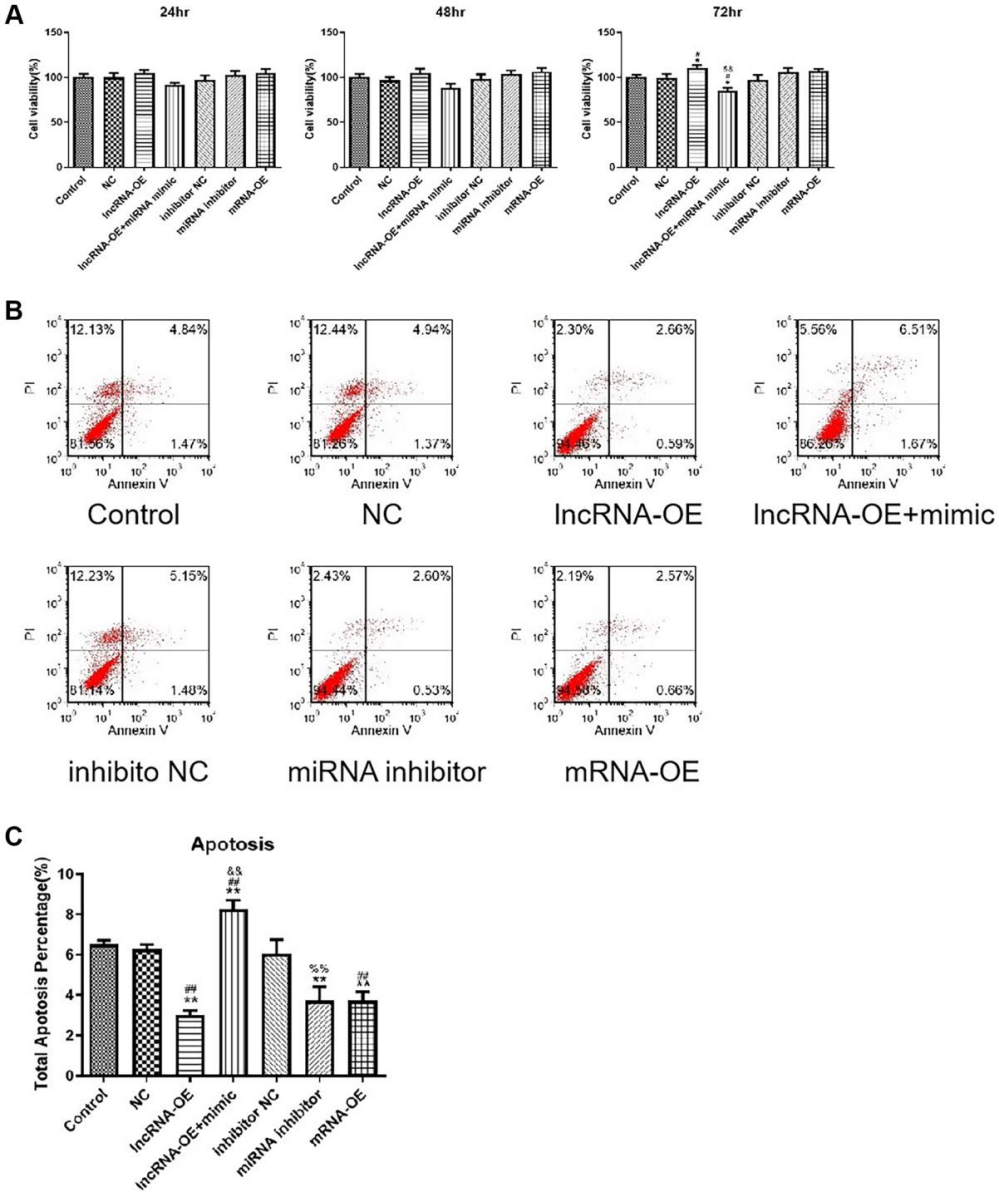
**LncRNA GPRC5D-AS1 regulated cell viability and cell apoptosis by miR-520d-5p.** (**A**) Cell viability was assessed by CCK-8 assay. 15 mM Dex was added in HSMM to establish atrophy cell model (control group). Empty plasmid (NC group), GPRC5D-AS1-OE (lncRNA-OE group), GPRC5D-AS1-OE + miR-520d-5p mimic (lncRNA-OE + mimic group), miRNA inhibitor control (inhibitor NC group), miR-520d-5p inhibitor (miRNA inhibitor group) or MYOD1-OE plasmid (mRNA-OE group) was transfected into atrophy cell model and incubated for 24 h, 48 h and 72 h. ^*^P < 0.05 compared with control group; ^#^*P* < 0.05 compared with NC group; ^&^*P* < 0.05, ^&&^*P* < 0.01 compared with lncRNA-OE group. (**B**) Cell apoptosis was assessed by flow cytometry. Groups were set as previously mentioned. Six different plasmids were transfected into atrophy cell model and incubated for 48 h. (**C**) Quantitative analysis of cell apoptosis. ^*^P < 0.05, ^**^P < 0.01 compared with control group; ^#^*P* < 0.05, ^##^*P* < 0.01 compared with NC group; ^&^*P* < 0.05, ^&&^*P* < 0.01 compared with lncRNA-OE group; ^%^*P* < 0.05, ^%%^*P* < 0.01 compared with inhibitor NC group.

Next, we further examined the effects of GPRC5D-AS1-mediated miR-520d-5p on atrophy cell viability and apoptosis. The CCK-8 assay revealed that overexpression of GPRC5D-AS1 increased the cell activity, and the addition of miR-520d-5p mimic reversed the phenomenon, making the cell activity significantly lower than the control group at 72 hours. In addition, miR-520d-5p inhibitor and MYOD1 overexpression could also mildly enhance cell activity. The results above indicated that GPRC5D-AS1 enhanced cell activity by inhibiting miR-520d-5p. This regulation may be related to the expression of MYOD1 (Figure 6A).

Apoptosis assay showed that GPRC5D-AS1 overexpression, miR-520d-5p inhibition or MYOD1 mRNA overexpression all significantly inhibited apoptosis; miR-520d-5p mimic had a significant negative effect on apoptosis inhibition in GPRC5D-AS1 overexpression group, and finally induced a significant increase in cell apoptosis (Figure 6B). The quantitative analysis of the results of cell apoptosis is shown in Figure 6C.

## DISCUSSION

Skeletal muscle has been recognized as a primary target tissue in the context of aging and aging-related diseases. Sarcopenia is one of the most common age-related conditions. As a result, it is necessary to investigate the molecular mechanisms by which skeletal muscle regulatory factor controls age-related pathologies in order to combat the problem in a fast-aging society.

lncRNAs have emerged as a novel class of regulators in skeletal muscle physiology [36–38]. Recent transcriptome analyses on a global scale have revealed a multitude of lncRNAs that play significant roles in the regulation of skeletal muscle formation and differentiation, underscoring the importance of lncRNAs in the process of myogenesis [3]. Dysregulated expression of lncRNAs has been observed in various muscular disorders, including sarcopenia [39]. Further research is required to clarify the molecular mechanisms of non-coding RNAs underlying aging-related sarcopenia, to better understand their huge potential as therapeutic targets and biomarkers for sarcopenia. Our previous experimental findings have provided evidence for the essential involvement of lncRNAs in the regulation of skeletal muscle atrophy and indicated three candidate lncRNAs [2]. As a result, lncRNA GPRC5D-AS1 is selected in the present study as the target gene to further study its effect on skeletal muscle aging.

Cell proliferation inhibition usually results from cell cycle arrest, among the G2 arrest has a pivotal role in senescence [40, 41]. While doing cell cycle analysis on three different lines of normal human fibroblasts, Zhiyong Mao et al. observe that a large fraction of senescent cell population is arrested in G2 [42]. Bortezomib inhibits C2C12 growth by G2/M phase cell cycle arrest and apoptosis [43]. The data by Jun-Hui Song et al. demonstrate that Bisphenol A inhibits cell proliferation by inducing G2/M cell cycle arrest via the ATM-CHK1/CHK2–CDC25c-CDC2 signaling pathway [44]. Our data indicated that a significant number in the atrophied skeletal muscle myoblasts of cells were arrested at G2/M phase, and overexpression of GPRC5D-AS1 could promote cell proliferation by decreasing G2/M phase arrest. Considering that apoptosis of skeletal muscle cells is believed to be a contributing factor to muscle atrophy [45], we hypothesized that GPRC5D-AS1 may exert an inhibitory effect on the apoptosis of skeletal muscle cells. This hypothesis was subsequently validated. When GPRC5D-AS1 was overexpressed in atrophy cells, cell viability increased and cell apoptosis decreased. On the other hand, proliferation is always associated with induction of apoptosis [46]. A study finds that lncRNA CRNDE promotes cell proliferation owing to the inhibition of apoptosis in hepatocellular carcinoma [47]. The role of lncRNA ROR in enhancing cell viability and proliferation, as well as inhibiting apoptosis, has been documented in esophageal squamous cell carcinoma cells and papillary thyroidal carcinoma cells [48, 49]. Thus, we speculate that GPRC5D-AS1 may promote cell proliferation through the apoptosis pathway or cell cycle pathway. However, the detailed mechanisms and signaling processes remain to be elucidated.

The *in vitro* experiments in this study have shown that the overexpression of GPRC5D-AS1 led to a significant increase in various muscle regulatory factors expression levels, including Myf5, Mef2c, MyoD and MyoG. These factors are mainly involved in the differentiation, proliferation, and fusion of myoblasts. Among these factors, MyoG plays a crucial role in the growth, development and regeneration of skeletal muscle [50, 51]. Additionally, Mef2c is a member of the myocyte enhancer factor 2 (Mef2) family, which involves in regulating the expression of muscle regulatory factors and skeletal muscle-specific transcription of these factors [52].

MyoD is widely recognized as the principal regulator of myogenic differentiation, as it facilitates the transcription of the MyoG and MEF2C genes, thereby stimulating the expression of muscle-specific genes and ultimately leading to the formation of myotubes [53–55]. Interestingly, Myf5 and MyoD are both co-expressed and bound to the same gene sites, yet they possess distinct functions [56]. Specifically, MyoD exhibits a greater capacity to recruit Pol II to bind the promoter of downstream genes. In this experiment, MYOD1 overexpression also led to upregulation of MyoG and Mef2C but not Myf5, in line with previous studies. The results indicated that GPRC5D-AS1 had a positive influence on skeletal muscle aging. Overexpression of GPRC5D-AS1 remarkably promoted proliferation and differentiation of skeletal muscle myoblasts, suggesting that GPRC5D-AS1 positively regulates skeletal muscle development.

Next, through the utilization of bioinformatics analysis and luciferase reporter vector assays, we predicted possible miRNAs that may bind GPRC5D-AS1 and demonstrated miR-520d-5p can be directly targeted by GPRC5D-AS1, that targets muscle regulatory factors to negatively modulate myoblast proliferation and differentiation. We observed the inter-regulation between GPRC5D-AS1 and miR-520d-5p, which were in agreement with the studies mentioned in the literature: one of molecular mechanisms by which cytoplasmic lncRNAs regulate gene expression is to interact with miRNA that bind directly to miRNA response elements (MREs) and function to control the availability of miRNA for binding to their target mRNAs [57–59]. RNA transcripts that possess miRNA-binding sites have the ability to interact and regulate one another by competitively binding to shared miRNAs, thereby functioning as competing endogenous RNAs (ceRNAs) [60, 61]. Numerous instances have already been observed where lncRNAs serve as ceRNAs for miRNAs. For instance, a muscle-specific lncRNA called lincMD1 sequesters miR-135 and miR-133, effectively modulating the expression of MEF2C and MAML1 mRNAs, respectively [62]. LincMD1 becomes activated during myoblast differentiation and exerts control over muscle differentiation in both human and mouse myoblasts through its ceRNA activity. In a separate study, it is found that lncARSR facilitates the expression of AXL and c-MET by competitively binding miR-34/miR-449, thereby enhancing resistance to sunitinib in renal cell carcinoma cells [63]. We found an interaction among GPRC5D-AS1 and miR-520d-5p, and validated GPRC5D-AS1 in dexamethasone-induced human muscle atrophy cell model, functions as ceRNA for miR-520-5d to promote skeletal muscle proliferation and differentiation. Our data support the notion that lncRNAs function as miRNA sponge, leading to a decrease in miRNA levels in the body and subsequently reducing miRNA’s inhibitory effect on downstream targets [64, 65].

In addition to their roles in human development, GPRC5D-AS1 and miR-520d-5p have been implicated in various cancers. For example, miR-520d-5p has been shown to promote chondrogenesis and regulate chondrocyte metabolic activities by targeting HDAC1 [66]. Given its anti-tumor effect, miR-520d-5p suppresses the proliferation and invasion of cervical cancer cells by regulating PTK2 [67]. In triple-negative breast cancer, PITPNA-AS1 upregulates SIK2 to exert oncogenic function through miR-520d-5p and DDX54 [68]. One study reported that miR-520d-5p can reduce the mutations in hepatoma cancer cells and human induced pluripotent stem cells-derivatives, which is regulated by nucleotide mutations in these cells [69]. On the other hand, GPRC5D-AS1 is recognized as prognostic marker for lung squamous carcinoma based on bioinformatics analysis [70, 71]. Our study enriches the knowledge of the function of this lncRNA and miRNA.

Currently, most studies on muscle atrophy have used aged mouse models or cells to date, but whether human counterparts have similar molecular functions in muscle mass remains unclear [72]. Hence, the potential applicability of these investigated factors for the treatment of muscle atrophy in humans should be carefully evaluated. We use the dexamethasone-induced human muscle atrophy cell model, which is relatively rare. In fact, the metabolic alterations caused by dexamethasone in cellular systems exhibit resemblances to the metabolic changes observed in muscle atrophy in both human pathological conditions and animal models [73]. There is suggestive evidence that the administration of dexamethasone does not lead to a decline in anabolic responsiveness, but rather induces an atrophic effect primarily by suppressing the basal synthesis of protein in myotubes [74]. This underscores the utility of this model for investigating potential strategies to mitigate muscle atrophy.

However, the interaction *in vivo* between GPRC5D-AS1 and miR-520d-5p in skeletal muscle aging is unknown. Animal model experiments are needed to further explore their roles in skeletal muscle. Furthermore, we observed a slight enhancement in the expression of GPRC5D-AS1 and a mild decrease in the expression of miR-520d-5p when MYOD1 was overexpressed. To date, we found no relevant literature on this interaction. Yiwen Guo et al. employ lncRNA and mRNA microarray analysis to ascertain 997 differentially expressed lncRNAs and 1,817 differentially expressed mRNAs, which are regulated by MyoD in muscle cells [75]. The functional predictions indicate that the majority of these lncRNAs are implicated in biological pathways associated with muscle differentiation and the cell cycle, along with co-expressed genes. We speculate that MYOD1 possible feeds back regulation of GPRC5D-AS1 and miR-520d-5p, which may be related to MYOD1 downstream factors; however, this requires further investigation.

In summary, this study elucidates the role and mechanism of lncRNA GPRC5D-AS1 in inhibiting muscle aging. Functionally, GPRC5D-AS1 enhances the differentiation and proliferation of myoblasts, while suppressing cell apoptosis *in vitro*. Mechanistically, GPRC5D-AS1 acts as a ceRNA for miR-520d-5p, leading to the inhibition of cell apoptosis and the regulation of muscle regulatory factors such as MyoG, Myf5, Mef2c, and MyoD. This subsequently promotes the proliferation and differentiation of myoblasts, thereby facilitating skeletal muscle development. Consequently, the identification and functional characterization of GPRC5D-AS1 in relation to muscle aging of this study holds great promise for the development of novel therapeutic interventions. LncRNA GPRC5D-AS1 may offer a potential therapeutic strategy for the treatment of age-related sarcopenia.

## MATERIALS AND METHODS

### Cell culture and treatment

Human skeletal muscle myoblasts (Lonza Japan, Tokyo, Japan) were maintained in Dulbecco’s modified Eagle medium (DMEM) (Gibco BRL, Grand Island, NY, USA), supplemented with 10% (vol/vol) fetal bovine serum (Thermo Fisher Scientific, Waltham, MA, USA), and cultured at 37°C with 5% CO2. Differentiation into myotubes was induced using DMEM supplemented with 2% horse serum, 1% penicillin/streptomycin and 2% glutamine. During this experiment, the medium was changed every 48 hours. Following incubation with high-glucose DMEM for four days, dexamethasone at different concentrations (Dex, 5 mM, 10 mM and 15 mM) were added and cells were again incubated for 48 hours. The expression of MyoD by qRT-PCR was performed to confirm the success of model establishment.

### Cell transfection

A 24-well plate was seeded with 1 × 10^5^ skeletal muscle myoblasts per well. When the confluence reached 70% in plates and then transfected with 0.5 μg plasmid per well by 1 μL Lipofectamine 2000 (Thermo Fisher SCientific, USA). Empty plasmid and GPRC5D-AS1-OE plasmid were obtained from Ribobio Biotechnology (Guangzhou Ribobio Co., Ltd., Guangzhou, China). MiR-520d-5p mimic, inhibitor NC and miR-520d-5p inhibitor were obtained from Biotend Biotechnology (Shanghai Biotend Co., Ltd., Shanghai, China).

Skeletal muscle myoblasts were divided into four groups: control group (cells without any treatment), model group (cells treated with Dex), negative control (NC) group (model group cells transfected with empty plasmid) and lncRNA-OE group (model group cells transfected with GPRC5D-AS1-OE plasmid).

Skeletal muscle myoblasts were divided into seven groups: control group (cells treated with Dex), NC group (control group cells transfected with empty plasmid), lncRNA-OE group (control group cells transfected with GPRC5D-AS1-OE plasmid), lncRNA-OE + mimic group (control group cells transfected with GPRC5D-AS1-OE plasmid + miR-520d-5p mimic), inhibitor NC group (control group cells transfected with miRNA inhibitor control), miRNA inhibitor group (control group cells transfected with miR-520d-5p inhibitor), and mRNA-OE group (control group cells transfected with MYOD1-OE plasmid).

### Cell counting kit-8 (CCK-8) assay

A 96-well plate was seeded with 1 × 10^3^ skeletal muscle myoblasts per well, followed by the addition of 10 μL of 10% CCK-8 solution (Biyuntian, China) to each well. A two-hour incubation period was followed for the plates. A microplate reader (MK3, Thermo Fisher Scientific) was used to measure absorbance at 450 nm.

### Flow cytometry analysis

Cell apoptosis and cell cycle were measured using flow cytometry (FACSCalibur, BD Biosciences, San Jose, CA, USA). Cell apoptosis was quantitated using the Annexin V-FITC/Propidium Iodide (PI) apoptosis kit (BD bioscience, USA). In brief, skeletal muscle myoblasts at 48 hours post-transfection were centrifuged at 200 × g for 5 min and re-suspended in 1 × Binding buffer. 100 μL cell suspension was transferred into test tube, then 5 μL PI and annexin V-FITC were added to the mixture, and the cells were incubated for 15 min in the dark at room temperature (25°C). Lastly, the apoptotic cells were assessed using a flow cytometer within 1 hour. After transfection for 48 hours, skeletal muscle myoblasts were centrifuged at 200 × g 4°C for 5 min. The cells were harvested and washed, then fixed in ice-cold 70% alcohol at 4°C overnight. Subsequently, the samples were incubated with 100 μg/ml RNase A (Beyotime, China; ST578) at room temperature for 30 min and stained with 50 μg/ml PI (Biolegend, USA; 421301) at room temperature for 30 min. Cell cycle was tested on flow cytometry.

### Dual-luciferase reporter assays

Skeletal muscle myoblasts were cultured in 12-well plates. After 70%–80% confluence of the cells, 50 nM of the psiCHECK2-GPRC5D-AS1-WT luciferase vector or psiCHECK2-GPRC5D-AS1-MUT vector, as well as 50 nM of NC, miR-520d-5p mimic were transfected by using Lipofectamine™ 3000 Transfection Reagent. The cells were harvested after 24 hours of transfection, and luciferase assays were performed with the Dual-Luciferase Reporter Assay System (Beyotime; RG027). To account for differences in transfection efficiency, renilla luciferase activity for each sample was normalized to firefly luciferase expression.

### qRT-PCR analysis

Total RNA of each group was extracted by TRIzol reagent (Invitrogen, USA) according to the manufacturer’s instructions (TaKaRa, Dalian, China, Product code: 9109). Then cDNA was synthesized by reverse-transcription reaction, using PrimeScript™ RT Master Mix (Perfect Real Time) (TaKaRa, Product code: RR036A). qRT-PCR was conducted under the following conditions: 50.0°C for 3 min, 95.0°C for 3 min, and 40 cycles of 95.0°C for 10 s and 60.0°C for 30 s. After reaction, melting curve analysis was performed by heating the reaction mixture from 60 to 95°C at a rate of 0.5°C/10 second. Primer sequences were as shown in Table 1.

**Table 1:** The primer sequences for mRNAs, microRNAs and long non-coding RNAs (lncRNAs).

**Primers**	**Sequence (5′–3′)**
GPRC5D-AS1-F	GCTGTGTGAGAACTCCGTGT
GPRC5D-AS1-R	ACTATCAAAGGCAGGTCGGTG
MyoD-F	CGCCATCCGCTATATCGAGG
MyoD-R	CTGTAGTCCATCATGCCGTCG
MyoG-F	GGGGAAAACTACCTGCCTGTC
MyoG-R	AGGCGCTCGATGTACTGGAT
Mef2c-F	GAACGTAACAGACAGGTGACAT
Mef2c-R	CGGCTCGTTGTACTCCGTG
Myf5-F	AACCCTCAAGAGGTGTACCAC
Myf5-R	AGGACTGTTACATTCGGGCAT
Wnt5a-F	ATTCTTGGTGGTCGCTAGGTA
Wnt5a-R	CGCCTTCTCCGATGTACTGC
GAPDH-F	TGACAACTTTGGTATCGTGGAAGG
GAPDH-R	AGGCAGGGATGATGTTCTGGAGAG
hsa-miR-520d-5p-F	GGCCGGTGTTGAAACAATCT
hsa-miR-520d-5p-R	GTCGTATCCAGTGCAGGGTCCGAG GTATTCGCACTGGATACGACGAAAGG
hsa-miR-524-5p-F	CTACAAAGGGAAGCAC
hsa-miR-524-5p-R	GTCGTATCCAGTGCAGGGTCCGAG GTATTCGCACTGGATACGACGAGAAA
hsa-miR-153-3p-F	TTGCATAGTCACAAAA
hsa-miR-153-3p-R	GTCGTATCCAGTGCAGGGTCCGAG GTATTCGCACTGGATACGACGATCAC

### Western blot

Proteins were isolated with RIPA lysis buffer (Beyotime, Shanghai, China) after 48 hours of transfection. All protein sample concentration was determined by the BCA (Thermo Fisher Scientific, USA) method, followed by separation on SDS-PAGE. Then, protein was transferred to a polyvinylidene difluoride (PVDF) membrane (Millipore, USA), blocked with 5% skim milk and incubated with primary antibodies of anti-Mef2c (Cal. No. 10056-1-AP, Proteintech, USA; 1:1000), anti-MyoG (Cal. No. ab77232, Abcam, USA; 1:1000), anti-MyoD (Cal. No. 18943-1-AP, Proteintech, USA; 1:1000), anti-GAPDH (Cal. No. 10494-1-AP, Proteintech, USA; 1:1000), anti-Myf5 (Cal. No. ab125078, Abcam, USA; 1:1000) and overnight at 4°C. On the second day, horseradish Peroxidase conjugated goat anti-rabbit IgG (H+L) (Cal. No. 111-035-003, Jackson ImmunoResearch, USA) was added and incubated at 37°C for 2 hours. Chemiluminescence was developed by ECL system (Millipore, USA).

### Statistical analysis

All data represented the results of three independent experiments and were presented as the mean ± standard deviation (SDs). Experimental data were processed in GraphPad Prism 5 (GraphPad Software, San Diego, CA, USA). *P* < 0.05 represented statistical significance.

## Supplementary Materials

Supplementary Table 1
