# Supplementary material for: lncRNA GPRC5D-AS1 as a ceRNA inhibits skeletal muscle aging by regulating miR-520d-5p
Source: Aging (Albany NY). 2023 Dec 9;15(23):13980–97. doi: 10.18632/aging.205279 (PMC10756129; doi:10.18632/aging.205279)
Supplement: Supplementary Table 1 [file aging-15-205279-s001.pdf]

SUPPLEMENTARY TABLE

Supplementary Table 1. Summarized cell cycle data.

		Experiment 1 (%)	Experiment 2 (%)	Experiment 3 (%)	Mean (%)	SD
Control	G0/G1	59.29	60.81	58.54	59.5467	1.1566
	S	15.01	14.97	18.64	16.2067	2.1074
	G2/M	25.7	24.22	22.82	24.2467	1.4402
Model	G0/G1	54.05	54.55	54.14	54.2467	0.2665
	S	2.03	3.01	4.16	3.0667	1.0661
	G2/M	43.92	42.44	41.7	42.6867	1.1304
NC	G0/G1	53.29	55.93	55.26	54.8267	1.3723
	S	4.81	1.06	2.97	2.9467	1.8751
	G2/M	41.9	43.01	41.77	42.2267	0.6815
lncRNA-OE	G0/G1	61.51	63.02	63.07	62.5333	0.8866
	S	6.74	8.68	9.68	8.3667	1.4948
	G2/M	31.75	28.3	27.25	29.1000	2.3543
